# ASO Author Reflections: Intracavitary Radiofrequency Ablation as a Means to Deescalate Radiation in Patients with Breast Cancer

**DOI:** 10.1245/s10434-026-19437-z

**Published:** 2026-03-04

**Authors:** V. Suzanne Klimberg

**Affiliations:** Department of Surgery, Department of Breast Surgical Oncology, The University of Texas Medical Branch, Galveston, TX USA

Ongoing research is advancing new and improved techniques and treatments for breast cancer. Preserving a patient’s body image during diagnosis and treatment are the subjects of ongoing studies and has led to innovative less morbid techniques for breast cancer surgery.^1^ As breast surgery has minimized its approach, the cosmetics and pain of patients with breast cancer suffer from continued treatment with post-surgical partial and whole breast radiation. We know from the NSABP B-21 trial that only about 16% of patients with favorable breast cancer recur on endocrine therapy alone, which can be reduced to 4% with additional radiation.^2^ Yet, survival remains the same with long-term follow-up. Breast radiation may lead to overtreatment in nearly 90% of patients. More modern studies comparing breast conservation surgery (BCS) with BCS+XRT still show about a 1% per year increase in local recurrence.^3^ Radiation also adds a significant cost, as well as travel time, which can be a deterrent to receiving BCS for rural and poor patients and especially for those located in low- and middle-income countries (LMICs).

The importance of the recently published paper “Open excision followed by cavitary RFA alone – A phase II ABLATE multicenter registry trial of 242 breast cancer patients” by Gallagher and colleagues is that surgeons can offer complete same-day treatment of the breast with BCS and intracavitary ablation with equivalent recurrence outcomes compared with partial or whole breast irradiation.^4^ The multicenter phase II ABLATE trial shows that 44-month median follow-up with both ductal carcinoma in situ and invasive breast cancer with less than 3% local recurrence rate without radiation and builds on prior single-institution trials of Klimberg and colleagues.^5^ Of particular note is the fivefold increase in pain in radiated versus ablated patients.

The ABLATE study lays the groundwork for a multicenter randomized trial using improved easier-to-use RFA technology developed solely for this purpose. As with any new procedure, this trial will require surgeon training in proper patient selection and procedure performance. A majority of our patients in the USA have small stage I low-grade breast cancer, and will benefit from deescalation of radiation. Intracavitary ablation will improve access for rural and poor patients, improve clinical outcomes as well as limit morbidity and financial toxicity due to low cost, and require less visits to complete therapy.
